# Supplementary material for: Patients and Communities Shape Regional Health Research Priorities: A Participatory Study from South Tyrol, Italy
Source: Healthcare (Basel). 2025 Nov 4;13(21):2797. doi: 10.3390/healthcare13212797 (PMC12608851; doi:10.3390/healthcare13212797)
Supplement: Supplementary file 1 [file healthcare-13-02797-s001.zip › healthcare-3938847-supplementary.pdf]

Supplementary Materials

Table S1. Questionnaire– shaping research together.

Dear participant,
This questionnaire is being conducted by the Institute of General Medicine and Public Health at Claudiana Bolzano. The aim is to gather your organization's views on important areas of research that are relevant to improving healthcare in South Tyrol. Your feedback will help to ensure that future research projects are more closely aligned with the actual needs of patients.
It takes about 15 minutes to complete. Thank you for your participation!

Name of your organization (optional)

Question	Answer options / format
Name of your organization (optional)	Short answer (text)

Which groups do you mainly represent? (Multiple selections possible)

Options
Older people
Children and young people
People with chronic illnesses
People with disabilities
Relatives and caregivers
People with mental illnesses
People from linguistically or culturally diverse backgrounds
General practitioners
Other (free text at the end of the questionnaire)

Your role in the organization

Options
Board member or management
Employee
Volunteer
Member or user
Family doctor
Other (free text at the end of the questionnaire)

Which of the following topics do you think should be given priority in health research in South Tyrol? (1 = no priority, 5 = highest priority)

Topic	1	2	3	4	5

Trustworthy health information and communication	☐	☐	☐	☐	☐
Recognition of frailty in old age and appropriate care	☐	☐	☐	☐	☐
Patient-centered palliative care and end-of-life care	☐	☐	☐	☐	☐
Mental health of children and adolescents	☐	☐	☐	☐	☐
Vaccine acceptance and culturally sensitive education	☐	☐	☐	☐	☐
Continuity and trust in primary care	☐	☐	☐	☐	☐
Environmentally friendly and safe prescription of medicines	☐	☐	☐	☐	☐
Access to healthcare for disadvantaged groups	☐	☐	☐	☐	☐
Health education in schools	☐	☐	☐	☐	☐
Effects of free choice of a trusted family doctor on waiting times for specialist appointments and diagnostic imaging (e.g., MRI, CT, ultrasound)	☐	☐	☐	☐	☐
Family consequences of different approaches to stress/psychological strain in men and women	☐	☐	☐	☐	☐

Other (free text at the end of the questionnaire)	☐	☐	☐	☐	☐

What unaddressed or urgent issues do you see in the local healthcare system?

Question	Answer options / format
What unaddressed or urgent problems do you see in the local healthcare system?	Long answer (free text)

Have your members or users ever been involved in planning or evaluating health services?

Options
Yes
No
Don't know

What barriers do your members experience in accessing healthcare?

Question	Answer options / format
What barriers do your members face in accessing healthcare?	Long answer (free text)

What would help your members feel more heard in healthcare decisions and research?

Question	Answer options / Format
What would help your members feel more heard in healthcare decisions and research?	Long answer (free text)

Is your organization interested in participating in research projects (e.g., on specific issues, workshops, exchange of experiences)?

Options
Yes
No
Maybe (more information requested)

How would you prefer to participate in setting priorities for research topics? (select max. 2)

Options / Opzioni
Online surveys
In-person or online workshops
Focus groups

Patient advisory board
Feedback on research results
Other (free text at the end of the questionnaire)

Would you like to add or emphasize anything else that is important from your organization's perspective—in particular, content that was not adequately covered in the previous selection fields (e.g., under "Other")?

Question	Answer options / format
Would you like to add or emphasize anything else that is important from your organization's perspective—in particular, content that was not adequately covered in the previous selection fields (e.g., under "Other")?	Long answer (free text)

Table S2. Codebook for inductively derived categories from open-text responses.

Domain	Code label	Brief description	Example [1]
Barriers to healthcare access	Waiting times and access	Delays in obtaining appointments or diagnostics; long waiting times in public system	"Appointments for MRI or specialist visits take months."
	Shortage of staff and physicians	Perceived lack of doctors, nurses, or specialists	"There are too few family doctors in rural areas."
	Bureaucracy and organization	Administrative hurdles, poor coordination, rigid procedures	"Too much paperwork and no one feels responsible."
	Inequalities and private sector	Unequal access between private and public care	"Those who pay privately get treated faster."
	Special or vulnerable groups	Barriers for people with disabilities, migrants, or low literacy	"People with disabilities find it hard to get to the hospital."
	Prevention and health promotion	Lack of preventive offers or follow-up	"There's too little health education and prevention."
	Human relationship and empathy	Lack of empathy, impersonal encounters	"Healthcare feels cold and impersonal."
	Aftercare for chronic diseases	Missing follow-up or coordination after hospital discharge	"After hospital discharge, no one checks on the patient."
	Digitalization and documentation	Difficulties with online systems or excessive digital paperwork	"Digital systems make it even more complicated."

	Governance and values	Leadership, communication, or system-level trust issues	"Health authorities should listen more to citizens."
Facilitators / Improvement suggestions ("to feel better heard")	Clearer communication	Transparent information, understandable language	"Use clearer and simpler information."
	More time for participation	Dedicated time for patient dialogue and feedback	"Take time to listen to patients properly."
	Active inclusion	Systematic involvement of patients or associations in decisions	"Include patients in planning meetings."
	Representation in governance bodies	Participation in formal committees or boards	"Create a patient advisory board at the hospital."
	Greater recognition of GPs	Stronger role for general practitioners in participation processes	"GPs should be involved more in decision-making."
	Better education and information	Educational initiatives and patient empowerment	"Offer more health literacy programs."
	Greater empathy from institutions	Institutional attitude change toward empathy	"Officials should show more empathy."
	Improved accessibility	Barrier-free infrastructure and materials	"Information should also be available for people with disabilities."
	Reduction of bureaucratic barriers	Simplify administrative processes	"Reduce unnecessary bureaucracy."
	Increased trust	Strengthen trust between institutions and the public	"Rebuild trust through honest communication."
Additional open-text themes (free comments)	System reform and governance	Calls for systemic or political reform	"The system needs a fundamental reorganization."
	Psychosocial support	Need for better mental health or family support	"Families under stress need more help."
	Vulnerable populations	Mentions of elderly, low-income, or marginalized groups	"Pay attention to those who are left behind."
	Prevention and health promotion	Calls for greater prevention efforts	"More prevention, less focus on cure."
	Appreciation and meta-comments	Positive remarks about the survey or project	"Thank you for giving us a voice."

[1] Translated from German.

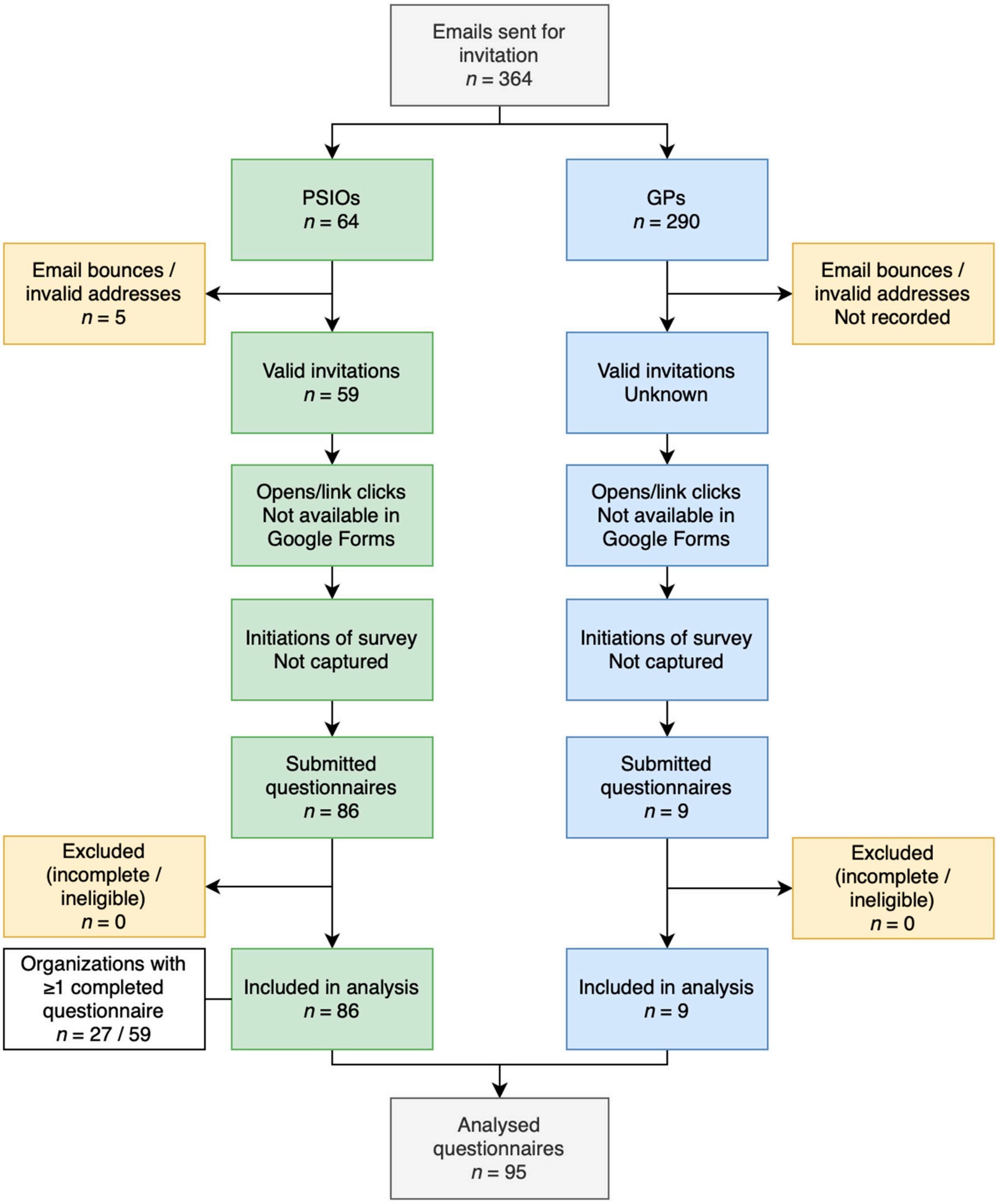

Figure S1. Flow diagram of email invitations, survey submissions, and analyzed questionnaires among patient and social interest organizations (PSIOs) and general practitioners (GPs).
